# Spondylodiscitis and spinal epidural abscess related to long‐term placement of an airway stent for malignant central airway obstruction

**DOI:** 10.1111/1759-7714.13530

**Published:** 2020-06-24

**Authors:** Kohei Shikano, Daisuke Ishii, Tomotaka Umimura, Shintaro Rakuman, Satoshi Maki, Hajime Kasai, Sumihisa Orita, Shunichiro Iwasawa, Toshihiko Sugiura, Seiji Ohtori, Koichiro Tatsumi

**Affiliations:** ^1^ Department of Respirology Graduate School of Medicine, Chiba University Chiba Japan; ^2^ Department of Orthopaedic Surgery, Graduate School of Medicine Chiba University Chiba Japan

**Keywords:** Airway obstruction, epidural abscess, lung cancer, self expandable metal stent

## Abstract

A 70‐year‐old male was referred to our hospital with lower limb muscle weakness and numbness of the left hand. The patient had previously been diagnosed seven years ago with lung cancer accompanied by central airway obstruction and had received chemoradiotherapy following placement of a metallic stent. Computed tomography (CT) scan revealed an osteolytic lesion which was adjacent to the fractured stent. T2‐weighted magnetic resonance imaging (MRI) demonstrated high signal intensity in the disc space. The patient was diagnosed with spondylodiscitis and spinal epidural abscess related to the airway stent. Despite hemilaminectomy, laminectomy and long‐term antibiotic therapy, the infection was uncontrolled. Moreover, osteolytic destruction and kyphotic deformity progressed. Removal of the airway stent was necessary; however, it was impossible because bronchial resection was required and the risk of mediastinal injury was considered to be high. The patient subsequently received palliative care. Long‐term airway stenting can cause spondylodiscitis and spinal epidural abscess. Indications for the placement of metallic stents for malignant central airway obstruction should be carefully evaluated after considering the difficulty in removal and the long‐term risk of severe complications.

**Key points:**

Significant findings of the study

Long‐term placement and fracture of the airway stent can cause spondylodiscitis and spinal epidural abscess.

What this study adds

The indication of placement of a metallic stent for malignant central airway obstruction should be considered with caution, especially if long‐term survival can be expected.

## Introduction

Malignant central airway obstruction (CAO) is a life‐threatening complication of lung cancer and often requires immediate treatment.^1^ Airway stenting is an important palliative treatment for CAO.^2^ It can improve symptoms and restore the performance status, thus facilitating chemotherapy and/or radiation therapy.^3^, ^4^ However, several stent‐related complications such as stent fracture and infection have been previously reported,^5^ but it remains undefined how complications in long‐term airway stenting for malignant CAO should be managed.

Here, we report a case involving a man who developed spondylodiscitis and spinal epidural abscess related to airway stent fracture seven years after airway stenting and chemoradiotherapy for lung cancer with CAO.

## Case report

A 70‐year‐old male was referred to our hospital complaining of lower limb muscle weakness and left‐hand numbness. The patient had been diagnosed with squamous cell lung cancer (cT4N2M0; stage IIIB) accompanied by CAO seven years previously, and following the placement of a self‐expandable metallic stent (Spiral Z; Medico's Hirata, Tokyo, Japan), had received chemoradiotherapy with cisplatin plus S‐1 and 59 Gy of thoracic radiation.

**Figure 1:**
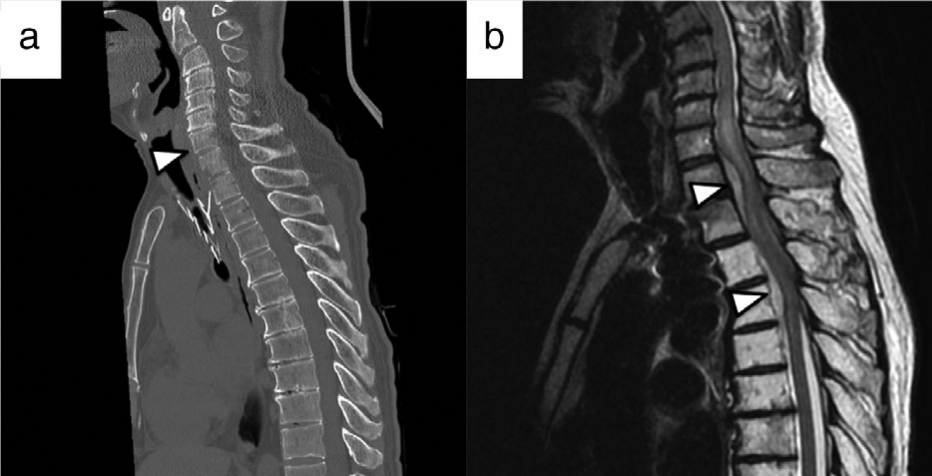
Imaging findings on admission. (**a**) Computed tomography (CT) scan revealed an osteolytic lesion at the T1–T2 disc level, adjacent to the fractured stent. (**b**) T2‐weighted magnetic resonance imaging demonstrated high signal intensity in the disc space, with an epidural abscess compressing the spinal cord.

On admission, blood examination revealed high levels of inflammatory markers. Computed tomography (CT) revealed an osteolytic lesion at the T1–T2 disc level, which was adjacent to the fractured airway stent. T2‐weighted magnetic resonance imaging (MRI) demonstrated high signal intensity in the disc space, with an epidural abscess compressing the spinal cord (Fig 1). The patient was finally diagnosed with spondylodiscitis and spinal epidural abscess related to the fractured airway stent.

**Figure 2:**
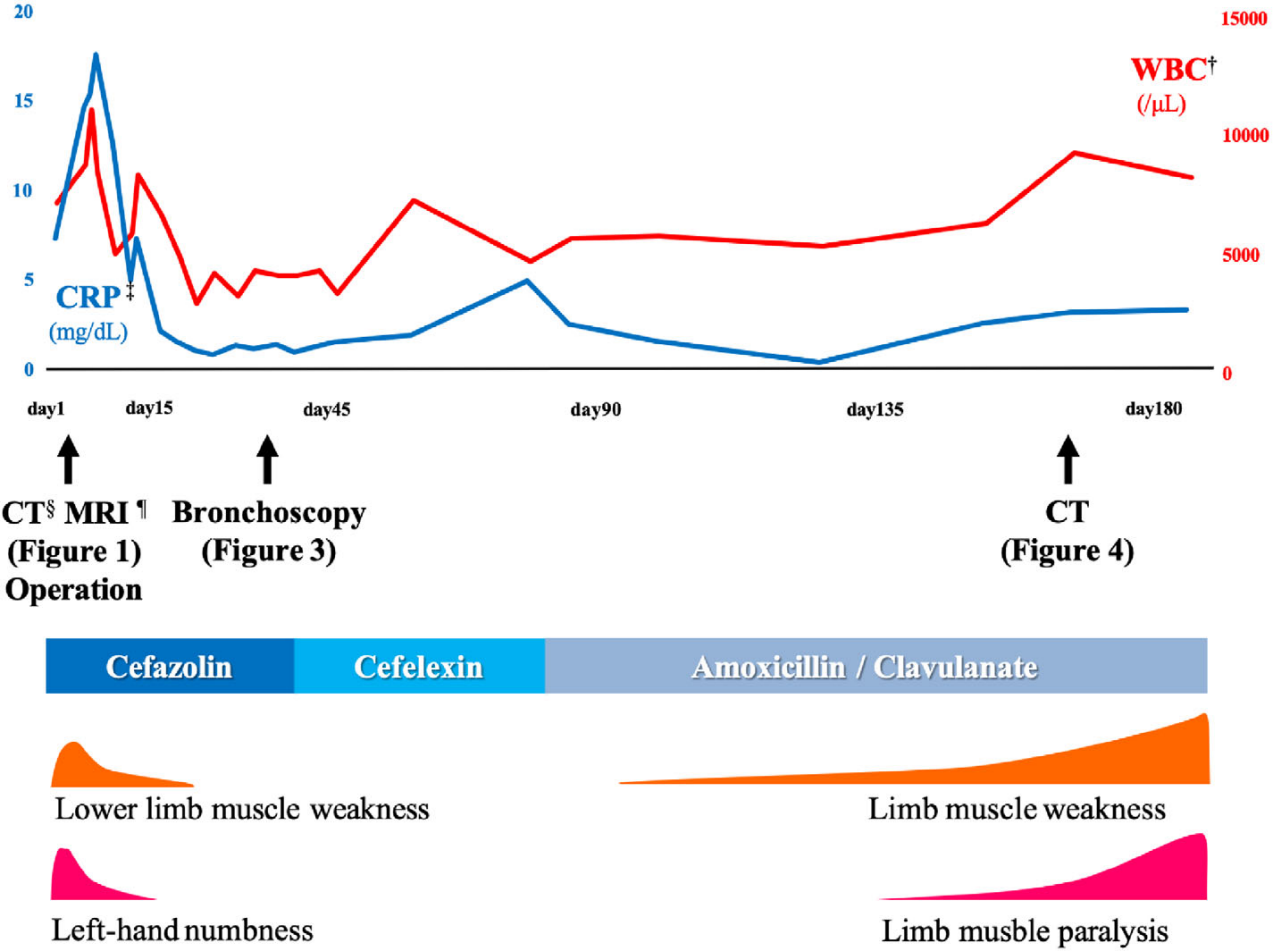
Clinical course of the patient. †WBC, White blood cell. ‡CRP, C‐reactive protein, §CT, computed tomography, ¶MRI, magnetic resonance imaging.

**Figure 3:**
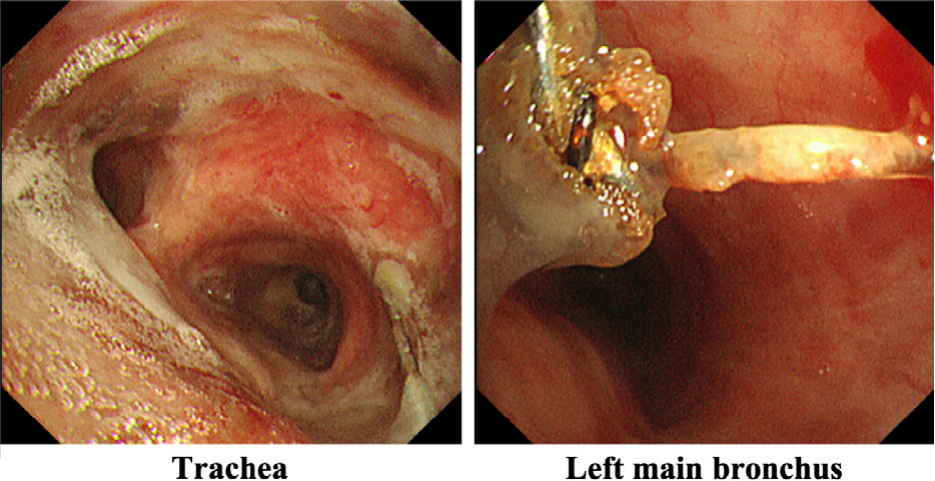
Bronchoscopy findings. Bronchoscopy showed the fractured airway stent covered with granulation tissue.

**Figure 4:**
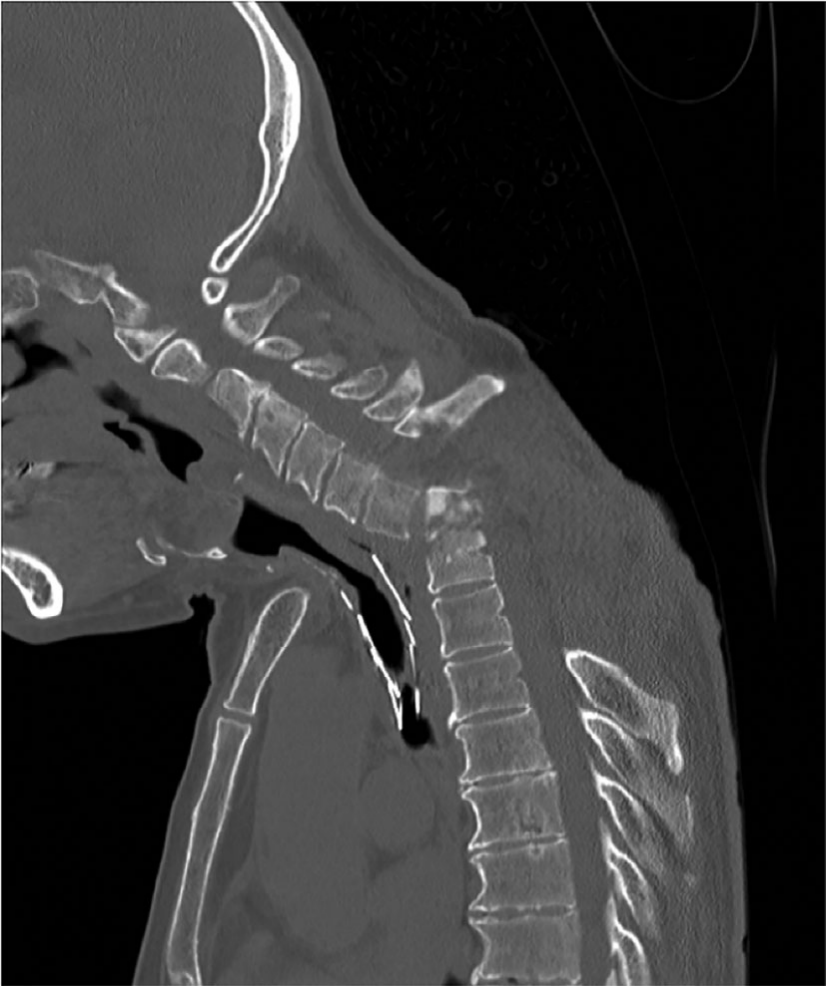
Computed tomography (CT) findings obtained on symptom recurrence. CTscan showed osteolytic destruction and kyphotic deformity at the T1–T2 level. The infection was uncontrolled and had spread to the surrounding areas.

The clinical course of the patient is summarized in Fig 2. Hemilaminectomy for C7 and laminectomy for T1–T4 were immediately performed for spinal cord decompression. Methicillin‐sensitive *Staphylococcus aureus* was isolated from the wound and blood cultures. Antibiotic therapy was initiated, and the patient's symptoms gradually ameliorated with a reduction in inflammatory markers. Removal of the airway stent for infection control was considered; however, bronchoscopy showed the fractured airway stent was covered with granulation tissue (Fig 3). Stent removal was considered impossible because bronchial resection was required and the risk of mediastinal injury was high. Thereafter, the patient received long‐term oral antibiotic therapy. However, inflammatory markers again increased. The neck pain recurred, and lower limb muscle weakness gradually progressed. CT showed osteolytic destruction and kyphotic deformity at T1–T2; this indicated uncontrolled infection that had spread to the surrounding area (Fig 4). Surgical site infection was inevitable without removal of the airway stent; however, this type of surgery is quite challenging. Following multidisciplinary discussion with the orthopedic surgery, general thoracic surgery, and respiratory medicine departments, we determined that the patient was not a good candidate for further surgical treatment. He primarily received palliative care with antibiotic therapy.

## Discussion

There were two important clinical issues found in this study. First, long‐term placement and fracture of airway stents can cause spondylodiscitis and spinal epidural abscess. Second, indications for airway metallic stenting should be considered with caution because of difficulty in removal and the long‐term risk of severe complications.

This is the first report of spondylodiscitis and spinal epidural abscess related to an airway stent. In a previous study, although 19% patients with airway stents experienced stent‐associated respiratory tract infection,^6^ there was no case of infection in tissues or organs other than the respiratory tract. Two cases of spinal epidural abscess related to esophageal stenting have been previously reported.^7^, ^8^ Erosion of the esophageal stent to the nearby vertebra in that study caused osteomyelitis and subsequent epidural abscess, and previous radiotherapy was the most important factor.^8^ The same mechanism may apply to our case that the airway stent fractured and eroded to the nearby vertebra after previous chemoradiotherapy.

Long‐term placement of metallic stents could cause several complications.^5^ Despite some reports describing the safe removal of airway stents,^9^, ^10^ metallic stent removal is challenging because of the potential risks of complications; retained stent pieces, mucosal tears with bleeding, reobstruction, and the need for postoperative mechanical ventilation.^11^ The U.S. Food and Drug Administration mentioned that the use of metallic stents as bridges to other therapies is not recommended.^12^ Complications of airway stenting for malignant CAO are rarely discussed because of the short life expectancy of patients. However, cases of long‐term survival after treatment for stage III or IV lung cancer are increasing because of advances in chemotherapy and chemoradiotherapy.^13^, ^14^ Thus, in cases of malignant CAO where long‐term survival is anticipated, tumor debulking instead of airway stenting should be performed in order to avoid stent‐related complications. If airway stenting is necessary, removable stents such as silicone or hybrid stents should be used. Although the risk of such complications with these removable stents are probably lower than with metallic stents, the stent should be removed as soon as the underlying problem is resolved. In our case, the metallic stent was placed as an emergency because of severe airway stenosis, indicating that there was no time to perform debulking. Moreover, hybrid stents were unavailable in Japan at that time. If airway stenting was avoided or a removable stent was placed and removed after chemoradiotherapy, the risk of complication was prevented. Regarding the metallic stent removal, bronchial resection was necessary in order to remove the stent extremely covered with granulation tissue, and severe complications were highly expected. As a result, the stent removal could not be performed even after chemoradiotherapy.

In conclusion, long‐term placement of metallic stents can cause spondylodiscitis and spinal epidural abscess. Indications for the placement of metallic stents for malignant CAO should be cautiously evaluated after considering the difficulty in removal and the long‐term risk of infection.

## Disclosure

The authors have no conflicts of interest.
